# Understanding Vaccine Hesitancy in Canada: Results of a Consultation Study by the Canadian Immunization Research Network

**DOI:** 10.1371/journal.pone.0156118

**Published:** 2016-06-03

**Authors:** Eve Dubé, Dominique Gagnon, Manale Ouakki, Julie A. Bettinger, Maryse Guay, Scott Halperin, Kumanan Wilson, Janice Graham, Holly O. Witteman, Shannon MacDonald, William Fisher, Laurence Monnais, Dat Tran, Arnaud Gagneur, Juliet Guichon, Vineet Saini, Jane M. Heffernan, Samantha Meyer, S. Michelle Driedger, Joshua Greenberg, Heather MacDougall, Canadian Immunization Research Network

**Affiliations:** 1 Département de médecine sociale et préventive, Université Laval, Québec, Québec, Canada; 2 Maladies infectieuses, Institut national de santé publique du Québec, Québec, Québec, Canada; 3 Maladies infectieuses et immunitaires, Centre de recherche du CHU de Québec–Université Laval, Québec, Québec, Canada; 4 Vaccine Evaluation Center, BC Children’s Hospital, and University of British Columbia, Vancouver, British Columbia, Canada; 5 Département des sciences de la santé communautaire, Université de Sherbrooke, Sherbrooke, Québec, Canada; 6 Department of Microbiology & Immunology, Dalhousie University, Halifax, Nova Scotia, Canada; 7 Clinical Epidemiology Program, Ottawa Hospital Research Institute, University of Ottawa, Ottawa, Ontario, Canada; 8 Department of Pediatrics, Dalhousie University, Halifax, Nova Scotia, Canada; 9 Département de médecine familiale et de médecine d’urgence, Université Laval, Québec, Québec, Canada; 10 Santé des populations et pratiques optimales en santé, Centre de recherche du CHU de Québec–Université Laval, Québec, Québec, Canada; 11 Nursing Faculty, University of Alberta, Edmonton, Alberta, Canada; 12 Department of Pediatrics, University of Calgary, Calgary, Alberta, Canada; 13 Department of Psychology, University of Western Ontario, London, Ontario, Canada; 14 Département d'Histoire, Université de Montréal, Montréal, Québec, Canada; 15 Division of Infectious Diseases, Hospital for Sick Children, Toronto, Ontario, Canada; 16 Département de pédiatrie, Service de néonatologie, Université de Sherbrooke, Sherbrooke, Québec, Canada; 17 Department of Community Health Sciences, University of Calgary, Calgary, Alberta, Canada; 18 Department of Production Animal Health, University of Calgary, Calgary, Alberta, Canada; 19 Alberta Health Services, Calgary, Alberta, Canada; 20 Department of Mathematics and Statistics, York University, Toronto, Ontario, Canada; 21 School of Public Health and Health Systems, University of Waterloo, Waterloo, Ontario, Canada; 22 Department of Community Health Sciences, University of Manitoba, Winnipeg, Manitoba, Canada; 23 School of Journalism and Communication, Carleton University, Ottawa, Ontario, Canada; 24 Department of History, University of Waterloo, Waterloo, Ontario, Canada; University College Cork, IRELAND

## Abstract

“Vaccine hesitancy” is a concept now frequently used in vaccination discourse. The increased popularity of this concept in both academic and public health circles is challenging previously held perspectives that individual vaccination attitudes and behaviours are a simple dichotomy of accept or reject. A consultation study was designed to assess the opinions of experts and health professionals concerning the definition, scope, and causes of vaccine hesitancy in Canada. We sent online surveys to two panels (1- vaccination experts and 2- front-line vaccine providers). Two questionnaires were completed by each panel, with data from the first questionnaire informing the development of questions for the second. Our participants defined vaccine hesitancy as an attitude (doubts, concerns) as well as a behaviour (refusing some / many vaccines, delaying vaccination). Our findings also indicate that both vaccine experts and front-line vaccine providers have the perception that vaccine rates have been declining and consider vaccine hesitancy an important issue to address in Canada. Diffusion of negative information online and lack of knowledge about vaccines were identified as the key causes of vaccine hesitancy by the participants. A common understanding of vaccine hesitancy among researchers, public health experts, policymakers and health care providers will better guide interventions that can more effectively address vaccine hesitancy within Canada.

## Introduction

Vaccination is widely considered to be one of the greatest achievements of public health [1]. Vaccination programs have contributed substantially to the decline in mortality and morbidity of infectious diseases of major public health significance [2]. To be successful in reducing the prevalence and incidence of vaccine-preventable diseases, vaccination programs rely on high and sustained vaccine uptake [3–5]. In addition to direct protection for vaccinated individuals, high vaccine coverage induces indirect protection for the overall community through the creation of herd immunity [6]. Childhood vaccination, moreover, is a specific public health priority because children are particularly vulnerable to infectious diseases. Despite the relatively high rate childhood vaccine coverage in Canada [7, 8], there are reasons to be concerned that vaccination programs might be losing public confidence [9, 10]. Recent outbreaks of vaccine-preventable diseases in North America and Europe have been linked to under-vaccinated or non-vaccinated communities, demonstrating the dramatic consequences of a decline in vaccine coverage [11]. For instance, in 2015, a large measles outbreak started by an unvaccinated traveler visiting Disneyland and spread to more than 20 US states, Mexico, and Canada [12].

“Vaccine hesitancy” is a concept now frequently used in vaccination discourse [13]. The increased popularity of this concept in both academic and public health circles is challenging previously held perspectives that individual vaccination attitudes and behaviours are a simple dichotomy of accept or reject. Rather, vaccine hesitancy, is defined, as a continuum of vaccine beliefs and associated behaviours ranging from complete refusal of all vaccines to complete vaccine acceptance [14, 15]. Vaccine-hesitant individuals are a heterogeneous group within this continuum [16, 17]. They may refuse some vaccines, but agree to others; they may delay or accept vaccines according to the recommended schedule but feel unsure about the “correctness” of their decision relative to their child’s health [17–19]. The World Health Organization (WHO) Strategic Advisory Group of Experts (SAGE) Working Group on Vaccine Hesitancy defined vaccine hesitancy as a “delay in acceptance or refusal of vaccines despite availability of vaccine services” [20]. According to SAGE, the scope of vaccine hesitancy includes instances where “vaccine acceptance in a specific setting is lower than would be expected, given the availability of vaccination services. Vaccine hesitancy is complex and multi-dimensional; it varies across time, place and vaccines” [20].

The WHO definition is focused on a binary behavioral outcome (e.g. vaccination or non vaccination) in contrast to definitions usually used in the literature which also include attitudes or beliefs (e.g. vaccination despite important doubts and concerns). Our research group identified a need for a common definition of vaccine hesitancy among researchers, public health experts, policymakers and health care providers to advance our theoretical understanding of the phenomenon and to better guide interventions that can more effectively address vaccine hesitancy within Canada [21].

In this context, the current study was designed to assess the opinions of researchers, public health experts, policy decision-makers and healthcare providers concerning the definition, scope, and causes of vaccine hesitancy in Canada.

## Materials and Methods

Our study used two rounds of stakeholder questionnaires in an approach based on the Delphi method, which is well suited for consensus-building [22, 23]. We sent online surveys (hosted by SimpleSurvey) (Saint-Jean-sur-Richelieu, Québec (QC)) to two stakeholder groups (hereafter named panels): 1) health professionals, researchers, experts and policy-makers who were members of the Canadian Association for Immunization Research and Evaluation (CAIRE) and of the Canadian Immunization Research Network (CIRN); and 2) front-line vaccine providers (nurses and physicians). Eligible participants (members of the research team were excluded) were invited to complete both questionnaires and two reminders were sent for the first and the second questionnaire. The research ethics committee at the *Centre de recherche du CHU de Québec–Université Laval* approved the study proposal.

### Study participants and recruitment

The panels were constructed using a purposive sampling technique in a two-step procedure. After obtaining the approval of the Canadian Immunization Research Network (CIRN) management committee and the Canadian Association for Immunization Research and Evaluation (CAIRE) administrators, the principal investigator (ED) sent a study invitation to both CIRN and CAIRE to distribute to all members inviting them to participate. CIRN[24] and CAIRE[25] are non-mutually exclusive pan-Canadian networks including, respectively, approximately 100 and 300 members with diverse occupations (policymakers, experts/scientists/researchers, health professionals) all involved in vaccination research, evaluation or decision-making, and sometimes vaccine administration. Within the two rounds of data collection from CIRN and CAIRE members (hereafter named “research networks members”), we identified a dearth of responses from front-line vaccine providers. To remedy to this, team members identified 10 to 12 experienced vaccine providers within their 5 respective provinces to be invited to participate. The data collection process for this second panel was similar to the first. However, we adapted the questionnaire to include a section regarding one-on-one counseling with vaccine-hesitant patients (e.g. How do you counsel patients who have doubts and concerns regarding vaccines? Are you reluctant to disclose information on risks of vaccination because of patients’ concerns and doubts?) and trust in research findings from different funding sources (e.g. government, industry).

### Data collection

Data were collected via questionnaires from each panel of participants (detail available in S1 Table). Questionnaires were available in French and in English. The questionnaire was first developed in English and then translated in French by the research team. A pre-test was made (for the first questionnaire) with both English and French-speaking participants. The final versions were revised to make sure they were identical. Two questionnaires were completed by each of the panels, ~ a month apart, with data from the first questionnaire informing the development of questions for the second. All potential participants were invited to respond to both questionnaires. The first questionnaire contained 15 open-ended questions to explore participants’ understanding of vaccine hesitancy and their views and perspectives about the causes and consequences of vaccine hesitancy. The questionnaire was developed on the basis of a previous study of the determinants of vaccine hesitancy conducted with vaccination programs managers from 13 countries [26]. The second questionnaire contained 16 closed-ended questions which asked participants to indicate their level of agreement, scored using a 10-point Likert scale, with statements about vaccine hesitancy that were derived from the first questionnaire. Participants were invited to provide additional comments in 3 open-text boxes in each section of the questionnaire. Both questionnaires also contained 5 questions to assess professional characteristics of the participants (region of practice, role in immunization, specialization, numbers of years of work in immunization, vaccine administration).

The e-mailed invitation included a description of the consultation’s purpose, the duration of the electronic survey (estimated to be less than 10 minutes), and a link to the online questionnaire.

### Data analysis

Content analysis was conducted on the open-ended responses. Conceptual categories were created based upon the themes addressed in the questionnaire and were updated and revised until no new properties, dimensions or relationships emerged. A senior research professional trained in qualitative methods (DG) coded the data; a second researcher (ED) reviewed this work. Participants’ definitions of vaccine hesitancy are illustrated in a word cloud that was developed using N’Vivo 10 software (all words mentioned by participants were used except for short articles and pronouns).

Descriptive statistics were generated for all closed-ended responses. Wilcoxon test or Fisher’s exact test, as appropriate, were used to compare the characteristics of participants between the first and second questionnaire rounds and validation analysis was conducted to identify any statistically significant differences in participants’ responses according to the province of practice. Responses on the 10-item Likert scale were divided into 3 subcategories (items 1 to 4 as “disagree”; 5 to 7 as “uncertain”; and items 8 to 10 as “agree”). Chi-squared or Fisher’s exact tests, as appropriate, were used to compare these responses to the respondent characteristics. In the second questionnaire, participants ranked the three main causes of vaccine hesitancy from a list of 15 items taken from responses to the first questionnaire. A score was assigned to the three items identified by each respondent, ranging from 1 (third most important cause) to 3 (first most important cause). A score of 0 was assigned to all items not selected among the three most important causes of vaccine hesitancy by the respondent. Item scores were summed to obtain a total raw score and means were calculated to have a final ranking of all causes. All statistical analyses were performed using SAS version 9.2 (SAS Inc., Cary, N.C., USA). In all instances a *P*-value <0.05 was considered statistically significant.

## Results

A total of 52 research networks members and 98 front-line vaccine providers completed the first questionnaire whereas 54 research networks members and 80 vaccine providers completed the second questionnaire. Professional and demographic characteristics of the participants in each round are presented in Table 1. Within each panel, there were no statistically significant differences in participants’ characteristics between the first and the second questionnaires.

**Table 1:** Demographics of participants in each questionnaire.*

	Research Networks members			Vaccine front-line providers		
	First questionnaire	Second questionnaire		First questionnaire	Second questionnaire	
	n (*%*)	n (*%*)	P-value	n (*%*)	n (*%*)	P-value
	(n = 52)	(n = 54)		(n = 98)	(n = 80)	
**Region of practice**						
Atlantic (New Brunswick, Nova Scotia, Newfoundland and Labrador, PEI)	3 (7)	9 (17)		7 (7)	6 (8)	
Québec	5 (12)	12 (23)		11 (11)	12 (15)	
Ontario	15 (37)	16 (31)	0.27	1 (1)	1 (1)	0.74
Prairies (Alberta, Manitoba, Saskatchewan)	13 (32)	12 (23)		8 (8)	3 (4)	
British Columbia	5 (12)	3 (6)		71 (72)	57 (72)	
**Vaccination practices**						
I administer vaccines myself	13 (32)	21 (42)	0.39	94 (98)	79 (100)	0.50
**Primary specialization**						
Epidemiologist	8 (19)	6 (11)				
Nurse	2 (5)	3 (6)		87 (89)	72 (91)	
Physician (family physician or paediatrician)	21 (51)	28 (54)	0.28	8 (8)	7 (9)	0.42
Program manager / administrator	5 (12)	2 (4)				
Other	5 (12)	13 (25)		3 (3)	0	
**Number of years of work in immunization**						
< 10 years	14 (35)	20 (38)		31 (32)	29 (37)	
10 to < 20 years	13 (32)	11 (21)	0.67	37 (38)	28 (35)	0.83
≥ 20 years	12 (30)	19 (36)		29 (30)	22 (28)	
Don't work in immunization	1 (2)	2 (4)				

*Missing answers for 11 research networks members in the first round and 1 vaccine provider in the second round

### Participants’ definition of vaccine hesitancy

In the first round of data collection, participants were asked how they would define “vaccine hesitancy”. The majority of participants defined vaccine hesitancy as a reluctance to receive (recommended) vaccinations, mainly due to concerns about safety and efficacy of vaccines. Others indicated that vaccine hesitancy was “having doubts” (some indicated *unjustified* doubts) with regards to vaccines.

In the second round, both stakeholder groups were invited to select their preferred definition among three choices provided: (a) the definition proposed by the WHO Strategic Advisory Group of Experts Working Group on Vaccine Hesitancy [20]; (b) a definition derived from highly cited studies on this topic [27, 28] and (c) a definition based on participants’ responses on the first questionnaire (Table 2). The third definition was preferred by the majority of participants in both panels.

**Table 2:** Participants’ preferred definition of vaccine hesitancy.

	Preferred definition	
Definitions of vaccine hesitancy	Research networks members	Vaccine Providers
	n (%)	n (%)
A) SAGE Working Group on Vaccine Hesitancy: *Vaccine hesitancy refers to delay in acceptance or refusal of vaccine despite availability of vaccination services*. *Vaccine hesitancy is complex and context specific*, *varying across time*, *place and vaccines*. *It is influenced by factors such as complacency*, *convenience and confidence*.	14 (28)	20 (27)
B) Definition derived from highly cited studies: *Vaccine attitudes can be seen on a continuum*, *ranging from total acceptance to complete refusal*. *Vaccine-hesitant individuals are a heterogeneous group in the middle of this continuum*. *Vaccine-hesitant individuals may refuse some vaccines*, *but agree to others; delay vaccines or accept vaccines but are unsure in doing so*.	14 (28)	12 (16)
C) Definition based on answers to the first round: *Vaccine hesitancy refers to reluctance to receive recommended vaccination because of concerns and doubts about vaccines that may or may not lead to delayed vaccination or refusal of one*, *many or all vaccines*.	22 (44)	41 (56)

Two missing answers from research networks members and 9 for vaccine providers.

### Participants’ views regarding the impact of vaccine hesitancy on vaccination programs in Canada

In the first round, 91% (n = 41) of research networks members reported that vaccine hesitancy had an impact on vaccination programs in Canada. These participants reported that low vaccination coverage and vaccination delays and refusals leading to vaccine-preventable diseases outbreaks were the most common results, along with increased time needed to allay public fears about vaccines.

Vaccine coverage is suboptimal, and vaccine providers waste time dealing with this that they could be spending on more productive things. (Research networks member, Ontario)

Many research networks members also noted that the impact of hesitancy on vaccination programs is hard to quantify due to the lack of routinely collected vaccination data in many provinces.

On-time immunization rates are almost certainly below what they were 10 years ago but with no vaccine registry, how are we to know? Vaccine hesitancy requires much more time from those who give vaccines to explain everything to parents. (Research networks member, Prairies)

In the first round of data collection, 87% (n = 85) of front-line vaccine providers reported that vaccine hesitancy resulted in increased time spent discussing vaccination issues with concerned patients. Extra appointments were needed to accommodate patients who wanted to spread out the vaccines over multiple visits.

More time needs to be taken with hesitant parents to provide information and education. No shows, cancellations and extra appointments for altered vaccine schedules affect workload and scheduling. Time spent following up with phone calls, letters, etc. to hesitant parents. Time spent trying to contact and offer immunization to those not immunized when doing a vaccine preventable disease follow-up. (Vaccine provider, Atlantic)

In the second round, 57% of research networks members and 75% of front-line vaccine providers agreed that vaccine hesitancy is a significant problem in Canada, and 76% of research networks members and 87% of vaccine providers agreed that it is contributing to sub-optimal vaccination coverage rates in Canada. The majority of research networks members (66%) and vaccine providers (78%) agreed that it is crucial to address this issue.

The second questionnaire asked the extent to which participants considered that vaccine hesitancy focused on specific vaccines. Measles-containing vaccines, newly introduced vaccines, influenza vaccines, and human papilloma virus (HPV) vaccines were deemed to contribute most to vaccine hesitancy by both groups of participants.

### Causes of vaccine hesitancy in Canada

In the first questionnaire, both panels of participants were asked about the main causes of vaccine hesitancy in Canada. The most frequently mentioned causes were misinformation or lack of knowledge and mistrust and fears around vaccination.

Misinformation about links between diseases, safety and immunizations. Educated, intelligent people who no longer take physician recommendations at face value and wish to research the recommendations on their own. (Research networks member, Ontario)

People not knowing the risks of disease, not seeing these diseases or burden of disease in their lifetime. (Vaccine-provider, Atlantic)

Some participants also mentioned that vaccine hesitancy is complex and that there are multiple causes.

There are many causes and these may differ by individual. Some may have fear, some may be political “conspiracy theories”, some may be true or perceived prior adverse events, some lack of knowledge, some beliefs “religious or other”. There is no one issue so there is no one answer or response.” (Research networks member, Atlantic)

In the second round of data collection, participants were asked to identify what they consider to be the main causes of vaccine hesitancy from a list of causes generated from responses to the first questionnaire (mean scores on the 10-point Likert scale are shown in Table 3). Participants were also asked to rank the three main causes of vaccine hesitancy. For both groups of participants, the diffusion of negative information on vaccination in Internet and social media followed by lack of knowledge about vaccination received the highest mean ranks. For research networks members and vaccine provider participants, respectively, mistrust in the pharmaceutical industry and a lack of confidence in vaccine safety, were the third most important cause of vaccine hesitancy.

**Table 3:** Causes of vaccine hesitancy in Canada.

	Research networks members		Vaccine providers	
	n	Mean Score*	n	Mean Score*
Lack of confidence in vaccines’ safety	53†	7.6†	79	8,5
Lack of confidence in vaccines’ effectiveness			79	6,8
Mistrust of the pharmaceutical industry	54	7.9	80	8,3
Mistrust of conventional medicine	54	5.9	80	6,4
Mistrust of the medical establishment	50	5.8	79	6
Diffusion of negative information on vaccination in Internet and social media	54	8.5	79	9,2
Preference for other mode of prevention	53	6.3	80	7,1
Lack of knowledge about vaccination, misinformation	54	8.4	79	8,7
Anti-vaccine movement and anti-vaccine lobby	54	7.7	77	8,3
Complacency	52	8	79	7,7
Lack of convenience	54	5.1	80	3,7
Issues related to vaccination policies and programs	54	5.8	80	5,6
Poor communication on vaccination by public health authorities	54	6	80	5,5
Religious beliefs against vaccination	54	5.1	80	4,5
Fear of needles and fear of pain	-	-	80	5

*Mean score on the 10-point Likert scale ranging from 1 = unimportant cause to 10 = very important cause

†Research networks members were asked one item: “*Lack of confidence in vaccines*”.

### Counseling vaccine-hesitant patients

Front-line vaccine providers were asked to answer specific questions regarding how they deal with vaccine hesitancy in their practice. In the first round of data collection, the majority of vaccine providers emphasized that they listen, try to understand the concerns of, and educate vaccine-hesitant patients about vaccines.

Reassure and listen. I answer parents’ questions and I’m honest and open to keep a trusting relation with parents. (Vaccine provider, Quebec)

Listen to the parent/client first to find out what their hesitancy is. Build a rapport. The reasons can vary greatly…Determine from this contact what the barriers are…it maybe misinformation and then can provide counsel along with written documentation. (Vaccine provider, Prairies)

The majority of vaccine providers (88%, n = 94) did not hesitate to disclose information on vaccination risks because of patients concerns and doubts. Most vaccine providers considered that it was their responsibility to ensure that patients understand the risks of both vaccines and of non-vaccination and that patients need to have all the facts to make an informed decision.

I feel confident in disclosing what the risks are and do so to everyone that I immunize. (Vaccine provider, Prairies)

I think it is better to fully inform people of all benefits and potential risks. I believe leaving out selective information would not only be unethical but could lead to a climate of distrust of health care providers and feed the irrational fears and beliefs that some people already have. (Vaccine provider, Atlantic)

In the second round, vaccine providers were asked about their level of agreement with the best practices in counselling vaccine-hesitant patients. The preferred approaches were to listen to concerns, to be non-judgemental and to correct misinformation (Table 4).

**Table 4:** Vaccine providers’ level of agreement with statements about the best ways to counsel vaccine-hesitant patients.

	Disagree	Somewhat Agree	Agree
	(1 to 4)	(5 to 7)	(8 to 10)
	%	%	%
Listen to the patients’ concerns, show reassurance, act and talk in a non judgmental way (n = 79)	0	4	96
Correct misinformation / provide most accurate information about vaccines (n = 79)	1	11	88
Remind of the benefits of vaccination and point out the risk of not immunizing (n = 79)	1	27	72
Give fact sheets and other resources about vaccination (e.g. websites, books) (n = 79)	4	32	64
Accommodate patients’ requests (e.g. alternative schedule, vaccine refusal) (n = 79)	4	33	63
Provide personal examples (own vaccination / examples of vaccine-preventable diseases in practice) (n = 78)	17	36	47
Refer patient to other providers or schedule another appointment to discuss vaccination concerns (n = 78)	26	33	41

In the second round, vaccine providers were asked about their level of confidence in dealing with vaccine-hesitant patients. Sixty-nine percent (69%) said they were comfortable dealing with vaccine-hesitant patients and 64% felt capable of counselling them. The majority of vaccine providers considered themselves to be well-prepared to provide information about risks and benefits, but fewer considered themselves well-prepared to discuss their patients’ values, priorities and goals, or the link between values and vaccination decisions (Table 5).

**Table 5:** Vaccine providers’ perceived preparedness in dealing with vaccine-hesitant patients (n = 79).

	Not at all prepared	Somewhat Prepared	Very prepared
	(1–2)	(3)	(4–5)
	%	%	%
How prepared are you to effectively provide information about risks and benefits of vaccination	2	11	87
How prepared are you to effectively discuss patient/family values, priorities and goals.	8	24	68
How prepared are you to effectively help patient/family understand the link between their values, priorities and goals and vaccinating/not vaccination (e.g., “I understand that it’s important to you to give your children the best possible chances of being healthy. Here is how that fits with vaccinating. . .”)	9	21	70

This question was based on a 5-point Likert-scale ranging from “Not at all prepared” to “Very prepared”.

Finally, vaccine-providers were asked about their level of trust in vaccine research funded by different research funding sources. Vaccine research funded by the government and by academic institutions was more highly trusted than industry funded research (Table 6).

**Table 6:** Vaccine providers’ level of trust in vaccine research based upon research funding sources (%).

	Disagree	Somewhat Agree	Agree
	(1 to 4)	(5 to 7)	(8 to 10)
	%	%	%
I trust findings when the research is funded by the government (Public Health Agency of Canada, provincial and territorial governments, etc.) (n = 98)	0	10	90
I trust findings when the research is funded by the private sector (pharmaceutical industries) (n = 96)	28	46	26
I trust findings when the research is funded by academic institutions (Canadian Institute of Health Research, Universities) (n = 96)	1	10	89
I trust findings when the research is funded by academic institutions in partnership with the private sector (pharmaceutical industries) (n = 96)	15	35	50

## Discussion

The aim of this study was to identify the views of Canadian vaccination experts and health professionals concerning the definition, scope, causes, and consequences of vaccine hesitancy in Canada. Our participants defined vaccine hesitancy as an attitude (doubts, concerns) as well as a behaviour (refusing some / many vaccines, delaying vaccination). Although both definitions are similar, this definition could be seen as broader than the definition adopted by the SAGE Working Group on Vaccine Hesitancy, which recognized vaccine hesitancy to be vaccination behaviour *per se* (delay in acceptance or refusal of vaccines). While the WHO definition refers to behavior, it also acknowledges that factors such as complacency, confidence and convenience can lead to vaccine hesitancy and these factors include beliefs, perceptions, attitudes and knowledge. The explicit recognition that attitudes and beliefs play an important role in influencing behaviour suggests aspects that could be addressed by public health interventions. For example, people who are “on the fence” in their attitudes and beliefs are an important group for which public health interventions are needed, because they are “at risk” of stopping vaccinating and may be more open to public health advice than the outright refusers [29, 30].

Our findings indicate that the majority of participants—both vaccine experts and front-line vaccine providers–have the perception that vaccine rates have been declining and consider vaccine hesitancy an important issue to address in Canada. In the absence of a pan-Canadian immunization registry linked with validated and standardized measures of vaccine hesitancy, we lack hard evidence to support an increase in the prevalence of vaccine hesitancy and its impact on vaccine uptake rates. However, a recent Ontarian study looked at trends in medical and nonmedical immunization exemptions to measles-containing vaccines over a decade. The authors found that the overall percentage of students with any exemption classification remained low between 2002/03 to 2012/13 (<2.5%). However, religious or conscientious exemptions significantly increased during the study period whereas medical exemptions significantly decreased for both 7- and 17 years old students [31]. Others studies conducted in the United States have also found hard evidence of an increase in nonmedical exemptions [32–34]. Furthermore, suboptimal vaccine uptake rates in Canada can be explained by barriers to vaccination in terms of ease of access to vaccination services. Indeed, at the population level, identifying, measuring and monitoring the proportion of individuals who are vaccine-hesitant but who still follow the recommended schedule is not a simple task. If vaccine hesitancy encompasses a heterogeneous group of individuals with diverse attitudes and behaviours, as we suggest, then operationalizing this concept will be challenging [35]. The concept of vaccine hesitancy has been criticized as being an “ambiguous notion with an uncertain theoretical background” [36]. As pointed out by Peretti-Watel and collaborators, the heterogeneity in the conceptualization is problematic. Two groups of people—those who are “uncertain but very interested and committed in vaccination issues are prone to information seeking and long and balanced decision-making”, and those who have “no definite opinion, little knowledge and little interest about vaccination issues and who randomly forget or delay some vaccines”—could both be considered vaccine-hesitant, while showing very different attitudinal and behavioural patterns [36]. Indeed, more effort is needed to improve the ability to measure and assess vaccine hesitancy at the population level. Because research has mainly focused on the metrics of vaccine uptake (coverage rates, delays, refusals), the degree to which vaccine hesitancy influences vaccination behaviours remains an important, though complex, domain for investigation [13]. There is an urgent need to develop good techniques to identify and monitor patterns of both “attitudinal” and “behavioural” vaccine hesitancy in individuals and populations, and over time [37]. The consensus for most questions found in the current study suggests a common conceptualization and could serve as a basis for the development of such techniques.

Our findings also illustrate common opinions among vaccine experts and stakeholders regarding the main causes of vaccine hesitancy in Canada. Negative and false information about vaccination online and in social media was perceived to be the most important cause of vaccine hesitancy by participants. Indeed, many studies have suggested that the ubiquity of anti-vaccination content on the Internet contributes to the increase in vaccine hesitancy [9, 38–43]. Most studies that have examined vaccination-related content on websites or social media platforms have shown that the quality of information is highly variable with a substantial volume of negative and inaccurate information [42, 44–50]. Despite the potential impact of the Internet on vaccine hesitancy, limited information is available about parental use of online vaccination information and its influence on their level of vaccine hesitancy and their decision-making regarding childhood vaccination [39, 51, 52]. Most studies are descriptive, and though many attribute the increase in vaccine hesitancy to negative vaccination-related content on the Internet, they offer limited empirical evidence to support these claims [39, 42, 44, 53]. The emergence of social media as a source of online health information concomitant with decreasing trust in vaccination signals a critical need to understand better the role of social media in vaccine hesitancy. Further, social media role in vaccine hesitancy creates a need to develop appropriate strategies for online communication; such strategies should aim to provide vaccine-supportive information, to address misinformation published online, and to correspond to parents’ needs and interests [45]. The perceived link between the sources of vaccine research (e.g., government versus industry) funding and trust or mistrust in vaccine information requires further research, especially in light of our participants’ own concerns regarding research funded by the industry.

According to participants, misinformation or lack of knowledge about vaccines are other important causes of vaccine hesitancy. Indeed, lack or inadequate knowledge is frequently raised by public health professionals who are dealing with vaccine-hesitant populations [54, 55]. Recent educational interventions to correct ‘misinformation’ about vaccines, however, were largely ineffective to reduce vaccine hesitancy and, even worst, contribute to augment negative attitudes in the most vaccine-hesitant participants [56, 57]. The “knowledge-deficit” assumption can lead to labelling parents with vaccination doubts as innumerate, irrational, emotional, or easily manipulated by anti-vaccination groups. This rationalist approach implies that decision-making about vaccination can be improved by “correcting” emotional, cognitive and social distortions or biases affecting judgement and that external influences, such as those triggered by media, can be offset [54, 58, 59]. Many studies, however, have shown that vaccine decision-making is complex and that knowledge is only one of the many determinants of vaccination decisions [35, 54, 60]. While vaccine hesitancy exists in all stratums of the population, it is often associated to highly-educated parents. Studies conducted in different settings have shown that non-compliant parents appear to be well-informed individuals who have considerable interest in health-related issues and actively seek information [61–63].

As our study has shown, most Canadian vaccine providers support listening to the concerns of vaccine-hesitant patients, reassuring them in a nonjudgmental way, and providing accurate information on vaccination [64, 65]. This is in contrast with the recent call for a “gloves off” approach by public health authorities in the midst of the 2015 measles outbreak [66]. Research shows the majority of patients see health care professionals as the most trusted source of information on vaccination [67, 68], and many tools and tips exist to help providers in their discussions with vaccine-hesitant or vaccine-refusing patients [69–72]. While approaches vary, they share common characteristics, such as the importance of maintaining a trustworthy patient-provider relationship, as well as tailoring communication to patients’ specific concerns and doubts. Three studies assessed the effects of partial or full patient decision aids, which are tools intended to complement discussions with health care professionals and to facilitate informed and values-congruent decisions. Few have shown measurable results [25, 73–75]. Clearly, our results showed providers recognized the common characteristics found in these approaches; however the lack of results from studied approaches indicates more research may be needed to identify and implement effective ways to support health care providers’ communication with vaccine-hesitant patients [64, 76].

The data from our study should be interpreted with some caveats. First, by design the results reported here represent the opinions of only some non-randomly selected key opinion leaders. The results of this study were not intended to be representative of all vaccination experts, health professionals and front-line vaccine providers in Canada. Moreover, the voluntary participant sample targeting individuals with vaccine expertise or front-line vaccine delivery experience resulted in selection bias towards individuals with high interest in the topic of vaccine hesitancy. In addition, studies have shown that front-line vaccine providers may themselves be vaccine-hesitant, thus unlikely to strongly recommend vaccines [77, 78]. We did not include specific questions vaccine providers’ own level of vaccine hesitancy and it is probable that participants in our study held pro-vaccine attitudes. However, one third of vaccine-providers who participate in our study felt uncomfortable dealing with vaccine-hesitant patients and inadequately prepared to counsel them. Further studies will be needed to better understand vaccine hesitancy among front-line vaccine providers. Moreover, despite having been invited, no key opinion leaders from the Northern territories participated in the study. Because we have adapted our questionnaire for the recruitment of vaccine providers, we were not able to regroup for analysis the responses of vaccine providers of both panels. Despite these caveats, our study has generated rich findings on the opinions of key stakeholders regarding the scope and impact of vaccine hesitancy in Canada. Because vaccine hesitancy is a relatively new research topic, the use of many open-ended questions allowed us to obtain the opinions of participants without biasing the responses based on the research team’s assumptions. The fact that all data were collected anonymously should also have minimized social desirability bias.

To conclude, this study has shown that vaccine hesitancy is a concern for Canadian vaccination experts and health professionals. In the context of declining trust in science and state institutions [79, 80] and increasing consumerist orientation to healthcare [81, 82], more and more people wish to be–and, indeed, are encouraged to be–engaged in health decisions and to feel empowered to do so [83–87], regardless of whether their sources of information are perceived by experts as lacking credibility. It is important for health professionals to recognize the impact of the broader social landscape that “gives shape to ideas and ideals” about health, prevention and what a good citizen does about vaccination [88].

## Supporting Information

S1 Author List Author List. (PDF)

S1 Table Questions asked during the First questionnaire and Second questionnaire. (PDF)
