# Supplementary material for: Understanding Vaccine Hesitancy in Canada: Results of a Consultation Study by the Canadian Immunization Research Network
Source: PLoS One. 2016 Jun 3;11(6):e0156118. doi: 10.1371/journal.pone.0156118 (PMC4892544; doi:10.1371/journal.pone.0156118)
Supplement: S1 Author List — (PDF) [file pone.0156118.s001.pdf]

SUPPORTING INFORMATION

S1. Author List of the Social Sciences and Humanities Network of the Canadian Immunization Research Network

Eve Dubé[1,2,3*], Dominique Gagnon[2], Manale Ouakki[2], Julie A. Bettinger[4], Maryse Guay[2,5], Scott Halperin[6], Kumanan Wilson[7], Janice Graham[8], Holly O. Witteman[9,10], Shannon MacDonald[11,12], William Fisher [13], Laurence Monnais[14], Dat Tran[15], Arnaud Gagneur[16], Juliet Guichon[17], Vineet Saini[18,19], Jane M. Heffernan[20], Samantha Meyer[21], S. Michelle Driedger[22], Joshua Greenberg [23], Heather MacDougall[24] on behalf of the Canadian Immunization Research Network[^]

[1] Département de médecine sociale et préventive, Université Laval, Québec, Québec, Canada

[2] Maladies infectieuses, Institut national de santé publique du Québec, Québec, Québec, Canada

[3] Maladies infectieuses et immunitaires, Centre de recherche du CHU de Québec – Université Laval, Québec, Québec, Canada

[4] Vaccine Evaluation Center, BC Children's Hospital, and University of British Columbia, Vancouver, British Columbia, Canada

[5] Département des sciences de la santé communautaire, Université de Sherbrooke, Sherbrooke, Québec, Canada

[6] Department of Microbiology & Immunology, Dalhousie University, Halifax, Nova Scotia, Canada

[7] Clinical Epidemiology Program, Ottawa Hospital Research Institute, University of Ottawa, Ottawa, Ontario, Canada

[8] Department of Pediatrics, Dalhousie University, Halifax, Nova Scotia, Canada

[9] Département de médecine familiale et de médecine d'urgence, Université Laval, Québec, Québec, Canada

[10] Santé des populations et pratiques optimales en santé, Centre de recherche du CHU de Québec – Université Laval, Québec, Québec, Canada

[11] Nursing Faculty, University of Alberta, Edmonton, Alberta, Canada

[12] Department of Pediatrics, University of Calgary, Calgary, Alberta, Canada

[13] Department of Psychology, University of Western Ontario, London, Ontario, Canada

[14] Département d'Histoire, Université de Montréal, Montréal, Québec, Canada

[15] Division of Infectious Diseases, Hospital for Sick Children, Toronto, Ontario, Canada

[16] Département de pédiatrie, Service de néonatologie, Université de Sherbrooke, Sherbrooke, Québec, Canada

[17] Department of Community Health Sciences, University of Calgary, Calgary, Alberta, Canada

[18] Department of Production Animal Health, University of Calgary, Calgary, Alberta, Canada

[19] Alberta Health Services, Calgary, Alberta, Canada

[20] Department of Mathematics and Statistics, York University, Toronto, Ontario, Canada

[21] School of Public Health and Health Systems, University of Waterloo, Waterloo, Ontario, Canada

[22] Department of Community Health Sciences, University of Manitoba, Winnipeg, Manitoba, Canada

[23] School of Journalism and Communication, Carleton University, Ottawa, Ontario, Canada

[24] Department of History, University of Waterloo, Waterloo, Ontario, Canada

^ Membership of the Canadian Immunization Research Network is provided on the internet network's website.
