# Supplementary material for: Understanding Vaccine Hesitancy in Canada: Results of a Consultation Study by the Canadian Immunization Research Network
Source: PLoS One. 2016 Jun 3;11(6):e0156118. doi: 10.1371/journal.pone.0156118 (PMC4892544; doi:10.1371/journal.pone.0156118)
Supplement: S1 Table — (PDF) [file pone.0156118.s002.pdf]

SUPPORTING INFORMATION

Table S1. Questions asked during the First questionnaire and Second questionnaire

First questionnaire - Research Networks members

- What do you think are the main issues or challenges faced by immunization programs at the moment? (open-ended question)
- Do you feel that there are more people having doubts and concerns regarding vaccines and immunization programs today than in the past? (Yes / No / Don't know)
- How would you define vaccine hesitancy? (open-ended question)
- Do you consider vaccine hesitancy to be an attitude (doubts, fears)? Or a behaviour (vaccine delays, vaccine refusal)? Please specify An attitude (open-ended question)
- Do you think vaccine hesitancy exists in Canada? (Yes / No)
  - If yes, what do you consider to be the causes of vaccine hesitancy in Canada? If no, why not?
  - If no, why not?
- Do you think vaccine hesitancy has any impact on vaccination programs in Canada? (Yes / No)
  - If yes, how and to what extent?
  - If no, why not?
- Is vaccine hesitancy focused in any specific geographic areas in Canada? (Yes / No)
  - If yes, in which geographic areas?
- Is vaccine hesitancy focused on specific vaccines in Canada? (Yes / No)
  - If yes, which vaccines?

- Is vaccine hesitancy focused on specific groups of people in Canada? (Yes / No)
  - If yes, in which groups of people?

Participant's characteristics

First questionnaire - Front-line providers

- What do you think are the main issues or challenges that you are dealing with as part of your work in immunization? (open-ended question)
- Do you feel that there are more people having doubts and concerns regarding vaccines and immunization programs today than in the past? (Yes / No / Don't know)
- How do you counsel patients who have doubts and concerns regarding vaccines? (open-ended question)
- Are you reluctant to disclose information on risks of vaccination because of patients' concerns and doubts? (Yes / No)
- How would you define vaccine hesitancy? (open-ended question)
- Do you consider vaccine hesitancy to be an attitude (doubts, fears)? Or a behaviour (vaccine delays, vaccine refusal)? Please specify An attitude (open-ended question)
- Do you encounter vaccine-hesitant patients in your practice? (Yes / No)
  - If yes, do you consider that vaccine-hesitant patients are sharing specific characteristics? What are these characteristics?
- What do you consider to be the causes of vaccine hesitancy? (open-ended question)
- Does vaccine hesitancy have any impact on your practice? (Yes / No)
  - If yes, how and to what extent?
  - If no, why not?

- Is vaccine hesitancy focused on specific vaccines in Canada? (Yes / No)
    - ▶ If yes, which vaccines?
- Please indicate the level of trust you have in research findings about vaccines and vaccination depending on the funding sources. (matrix of choices)

Participant's characteristics

Second questionnaire - Research Networks members

- Please indicate your level of agreement with the following statement: *It is crucial to address vaccine hesitancy in Canada.*
- Please indicate which one of the following vaccine hesitancy definitions should be used in Canada. (See **Table 2** for the definitions)
- Please indicate your level of agreement with the following statement: *Vaccine hesitancy is an attitude (such as doubts and fears) that could lead to behaviour (such as refusing or delaying vaccination).*
    - ▶ If you have additional comments on the preceding questions, please write them here.
- Do you consider these factors as important causes of vaccine hesitancy in Canada? (See **Table 3** for the causes)
    - ▶ What do you consider to be the three main causes of vaccine hesitancy in Canada? Please rank what you consider to be the three main causes of vaccine hesitancy (among causes presented in **Table 3**)
- If you have additional comments on the main causes of vaccine hesitancy, please write them here.
- Please indicate your level of agreement with these statements on the impact of vaccine hesitancy on immunization programs in Canada: *Vaccine hesitancy contributes to sub-optimal*

vaccination coverage rates in Canada / Vaccines hesitancy contributes to an increase in vaccine-preventable disease cases and leads to possible outbreaks / Vaccine hesitancy places an increased burden on health professionals to address concerns and answer questions of vaccine-hesitant patients / Vaccine hesitancy contributes to a reluctance of decision-makers to introduce new vaccines to the provincial schedules.

- Please indicate your level of agreement with the following statement: *It is difficult to quantify the impact of vaccine hesitancy on immunization programs because of insufficient evidence*
- Please indicate to what extent you consider vaccine hesitancy is focused on specific vaccines (matrix of choices).
- Do you consider that vaccine hesitancy in Canada is : *Clustered in specific groups / Diffused in the entire Canadian population / Both*
- Please indicate to what extent you consider that the following groups of people are more prone to be vaccine hesitant? (matrix of choices)

  ▶ If you have additional comments on the preceding questions, please write them here.

Participant's characteristics

Second questionnaire - Front-line providers

- Please indicate your level of agreement with the following statements: *Vaccine hesitancy is an important problem in Canada / It is crucial to address vaccine hesitancy in Canada / I am comfortable dealing with vaccine hesitant patients in my practice / I feel capable of counselling vaccine hesitant patients in my practice*
- Please indicate which one of the following vaccine hesitancy definitions should be used in Canada. (See **Table 2** for the definitions)
- Please indicate your level of agreement with the following statement: *Vaccine hesitancy is an attitude (such as doubts and fears) that could lead to behaviour (such as refusing or delaying*

vaccination).

- ▶ If you have additional comments on the preceding questions, please write them here.

- Do you consider these factors as important causes of vaccine hesitancy in Canada? (See **Table 3** for the causes)

  - ▶ What do you consider to be the three main causes of vaccine hesitancy in Canada? Please rank what you consider to be the three main causes of vaccine hesitancy (among causes presented in **Table 3**)

- If you have additional comments on the main causes of vaccine hesitancy, please write them here.

- Please indicate your level of agreement with these statements on the impact of vaccine hesitancy on immunization programs in Canada: *Vaccine hesitancy decreases vaccination coverage (and/or on-time vaccination) / Vaccines hesitancy requires more time during consultation to address patients' concerns and decreases the number of patients that can be seen / Vaccine hesitancy poses personal challenges for vaccine providers (feeling of incompetence in addressing patients' concerns, conflict with own beliefs about vaccines, concerns regarding patients' requests for alternative schedule, etc.) / Vaccine hesitancy results in difficulties when recommending and administering vaccines newly introduced in recommended schedule.*

- Please indicate your level of agreement with the following statements about the best ways to counsel vaccine hesitant patients. (See **Table 4** for the ways to counsel vaccine-hesitant patients)

- Please answer the following questions on a scale of 1 to 5 (1 = not at all prepared, 5 = very prepared) (See **Table 5** for the questions)

- Please indicate to what extent you consider vaccine hesitancy is focused on specific vaccines

(matrix of choices).

- Please indicate to what extent you consider that the following groups of people are more prone to be vaccine hesitant? (matrix of choices)

  - ▶ If you have additional comments on the preceding questions, please write them here.

Participant's characteristics
